# Brain Injuries Associated With Maxillofacial Injuries: A Retrospective Study

**DOI:** 10.7759/cureus.88958

**Published:** 2025-07-29

**Authors:** Vigneswaran T, Yokeshkumar P, Prabhusankar K, Priyadharsana PS, Effie Edsor, Vigneshwar S, Vignesh K

**Affiliations:** 1 Department of Oral and Maxillofacial Surgery, RVS Dental College and Hospital, Coimbatore, IND

**Keywords:** facial fractures, incidence, maxillofacial trauma, road traffic injuries, traumatic brain injuries

## Abstract

Objective: This study aims to quantify the prevalence and severity of brain injuries in patients with maxillofacial fractures admitted to our trauma center over an eight-month period. Additionally, it seeks to analyze the correlation between specific patterns of facial fractures and the occurrence of brain injuries to enhance diagnostic accuracy, treatment strategies, and patient outcomes.

Methods: A retrospective analysis of medical records from 220 patients who presented with maxillofacial injuries at our center between January 2024 and August 2024 was conducted. Patients with isolated facial fractures and those with associated brain injuries were identified. Clinical parameters, including age, gender, mechanism of injury, type of facial fractures, Glasgow Coma Scale (GCS) score at admission, radiological findings, and treatment outcomes, were reviewed.

Results: Out of 220 patients, 154 (70%) had documented brain injuries in addition to maxillofacial trauma. The most common mechanism of injury was motor vehicle accidents (45%), followed by falls (25%), interpersonal violence (20%), and sports and occupational injury (10%). Among the facial fractures, zygomatic fractures were most frequently associated with brain injuries (40%), followed by frontal bone fractures (30%) and mandibular fractures (15%). Patients with associated brain injuries had significantly lower GCS scores upon admission and higher rates of complications, including increased intracranial pressure and longer hospital stays. Surgical intervention for maxillofacial fractures was delayed in 25% of patients with brain injuries due to the need for neurosurgical management.

Conclusion: The findings of this study suggest a high correlation between certain types of facial fractures and the presence of brain injuries. Early identification of brain injuries in patients with maxillofacial trauma is crucial for optimizing patient outcomes. A multidisciplinary approach involving both maxillofacial surgeons and neurosurgeons is recommended for the management of these complex injuries.

## Introduction

Facial fractures frequently occur as a result of craniofacial trauma, commonly stemming from incidents such as motor vehicle accidents, falls, assaults, and injuries related to sports. Although these fractures can lead to significant functional and cosmetic issues, their relationship with traumatic brain injury (TBI) is a crucial yet often overlooked factor in patient care. Research indicates that facial fractures can act as both a sign and a potential safeguarding factor in head trauma, with some studies showing a strong link between midface and mandibular fractures and accompanying brain injuries [1,2].

Traumatic brain injury continues to be a significant public health concern, leading to considerable morbidity and mortality on a global scale. It is estimated that around 50% of individuals with facial fractures suffer from some level of TBI, which can vary from mild concussions to severe intracranial hemorrhages [3,4]. The mechanism of injury, distribution of force, and specific anatomical area affected are crucial factors influencing the severity of both the facial fracture and the related brain injury [5].

Orbital, zygomatic, and frontal bone fractures have been closely linked to intracranial trauma due to their anatomical proximity to the brain [6,7]. Fractures involving the mandible, on the other hand, have been debated as potential shock absorbers that may reduce the impact transmitted to the skull [8]. Despite these biomechanical considerations, multiple studies have shown that facial fractures do not necessarily prevent brain injury, and in many cases, patients sustain concurrent TBIs requiring urgent intervention [9].

The clinical presentation of facial fractures accompanied by brain injuries can vary significantly, often complicating the initial diagnosis and management [10]. Neurological signs such as loss of consciousness, confusion, and memory loss may be present alongside indications of facial trauma, highlighting the importance of a comprehensive neurological evaluation in these situations [11]. Advanced imaging methods, such as computed tomography (CT) and magnetic resonance imaging (MRI), are essential for detecting both bone injuries and underlying brain conditions [12].

Understanding the relationship between facial fractures and TBIs is essential for optimizing patient outcomes. Prompt recognition of brain injury in patients with facial fractures can lead to early interventions that mitigate long-term neurological deficits [13]. This article aims to explore the incidence, pathophysiology, diagnostic challenges, and management considerations of brain injury associated with facial fractures, providing insights into their complex interplay in trauma patients [14].

## Materials and methods

Study design and setting

This retrospective study was conducted at RVS Dental College and Hospital. The research focused on patients who presented with maxillofacial injuries over a defined period of eight months, spanning from January 2024 to August 2024. Patient data were retrieved and analyzed from hospital records, allowing for a comprehensive evaluation of the patterns, causes, and types of facial injuries encountered during this timeframe. The study aimed to gain insights into the demographic distribution, etiology, clinical presentation, and management approaches associated with maxillofacial trauma in this region, contributing valuable information to the existing body of literature on facial injury epidemiology.

Patient selection

A total of 220 patients with confirmed maxillofacial injuries were included in this retrospective analysis. These patients were identified through a review of hospital medical records and trauma databases maintained at RVS Dental College and Hospital. Both individuals with isolated maxillofacial trauma and those presenting with concomitant craniofacial or brain injuries were considered for inclusion. To ensure data reliability and consistency, specific exclusion criteria were applied: patients were excluded if their medical records were incomplete, if they had succumbed to their injuries prior to hospital admission, or if they were under the age of 18 years. This selection process ensured the inclusion of cases with adequate clinical documentation and relevance to the study's objectives.

Data collection

Demographic data, including patient age, sex, and the etiology of the injury, were systematically recorded for each case. In addition, comprehensive clinical information was collected to facilitate detailed analysis. This included the Glasgow Coma Scale (GCS) score assessed at the time of admission, the specific types and locations of maxillofacial fractures, the presence and nature of any concurrent brain injuries, radiological imaging findings, the treatment modalities employed, and overall patient outcomes. Maxillofacial fractures were classified based on anatomical location, with categories including zygomatic complex fractures, frontal bone fractures, mandibular fractures, orbital fractures, nasal bone fractures, and Le Fort I, II, and III fractures. Concurrent brain injuries were categorized according to computed tomography (CT) scan findings and included cerebral contusions, intracranial hemorrhages (such as epidural, subdural, and subarachnoid hemorrhages), skull fractures, and diffuse axonal injuries. This comprehensive categorization enabled a nuanced understanding of the relationship between facial trauma and associated intracranial injuries.

Statistical analysis

Statistical analysis was performed using IBM SPSS Statistics software version 25.0 (IBM Corp., Armonk, NY). Descriptive statistics were used to summarize demographic and clinical characteristics of the study population. The prevalence of brain injury among patients with different types of maxillofacial fractures was calculated to identify potential patterns and correlations. Associations between the types of facial fractures and clinical outcomes, including the presence of brain injuries, were evaluated using chi-square (χ²) tests for categorical variables. To further assess the strength of these associations and adjust for potential confounding variables, logistic regression models were employed. A p-value of less than 0.05 was considered to indicate statistical significance throughout the analysis.

## Results

Patient demographics and mechanism of injury

**Figure 1:**
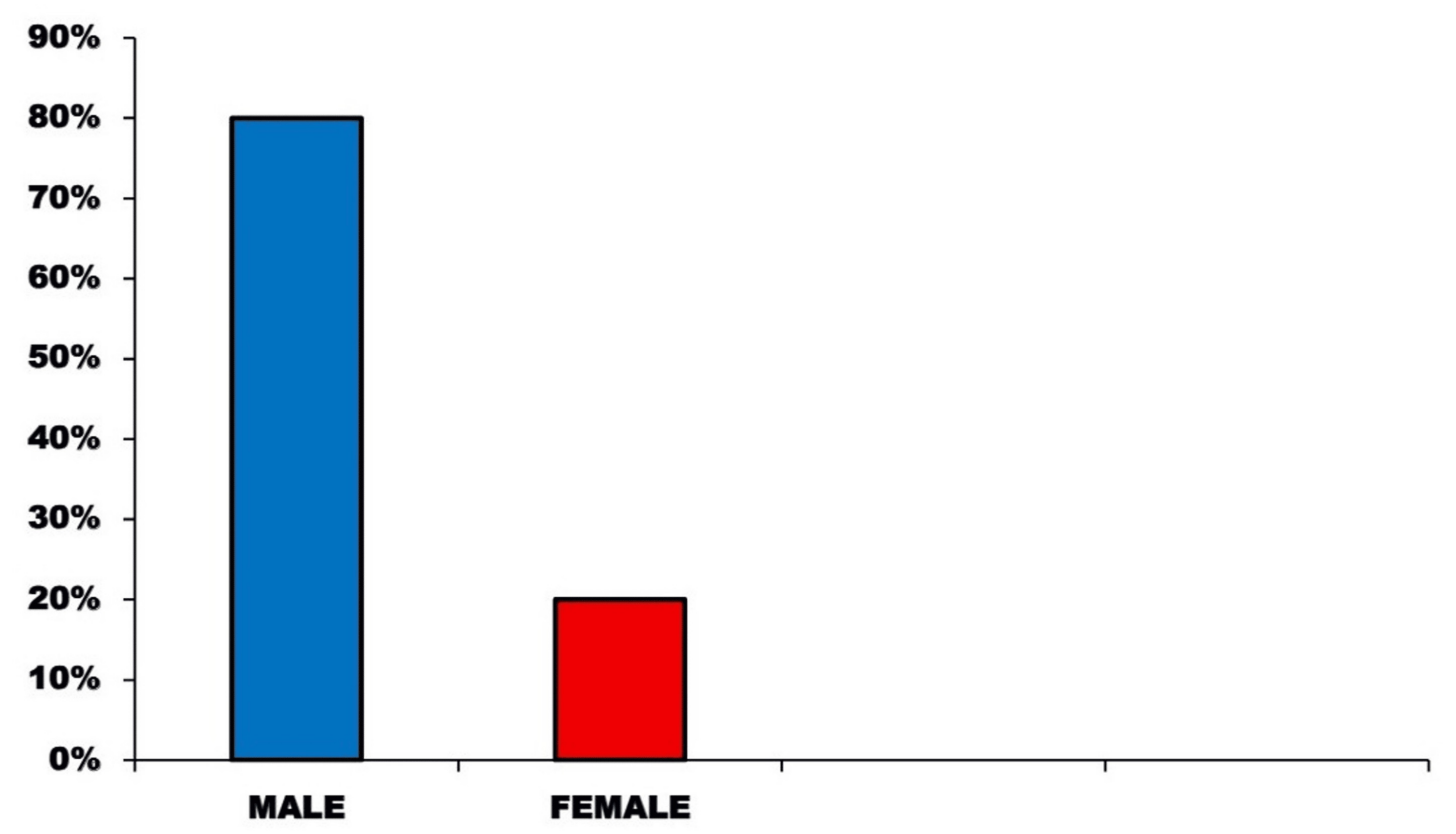
Distribution of head injuries according to sex

**Figure 2:**
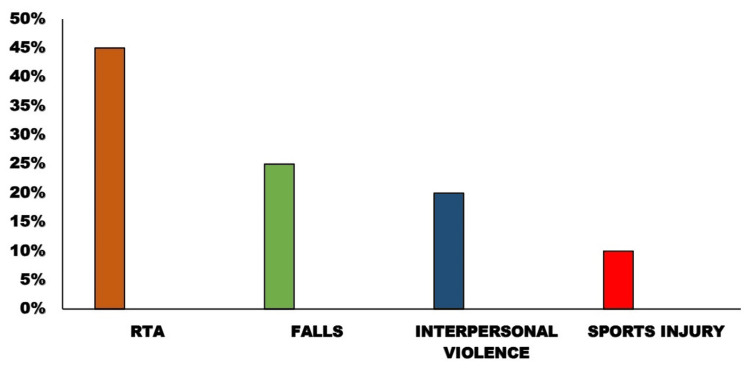
Distribution of head injuries according to etiology RTA: road traffic accident

In the study, 220 patients were involved, with a significant proportion being male (80%) and an average age of 34 years (age range: 18-65 years) (Figure 1). The primary causes of injury were motor vehicle accidents, which made up 45% of the cases, followed by falls at 25% and interpersonal violence at 20%. The remaining 10% of incidents were attributed to sports injuries and occupational accidents (Figure 2).

Prevalence of brain injury

**Figure 3:**
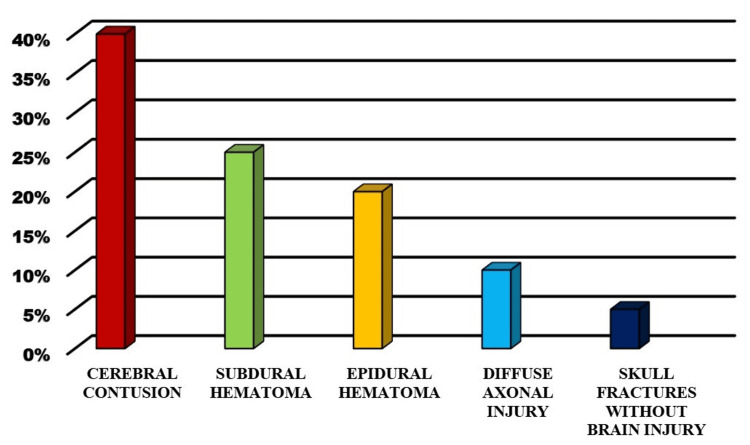
Different types of head injuries (in %)

Of the 220 patients with maxillofacial injuries, 154 (70%) had concomitant brain injuries. The most frequent types of brain injuries were cerebral contusions (40%), subdural hematomas (25%), and epidural hematomas (20%). Diffuse axonal injury was noted in 10% of patients, and 5% had skull fractures without significant brain injury (Figure 3).

Correlation between facial fractures and brain injuries

**Figure 4:**
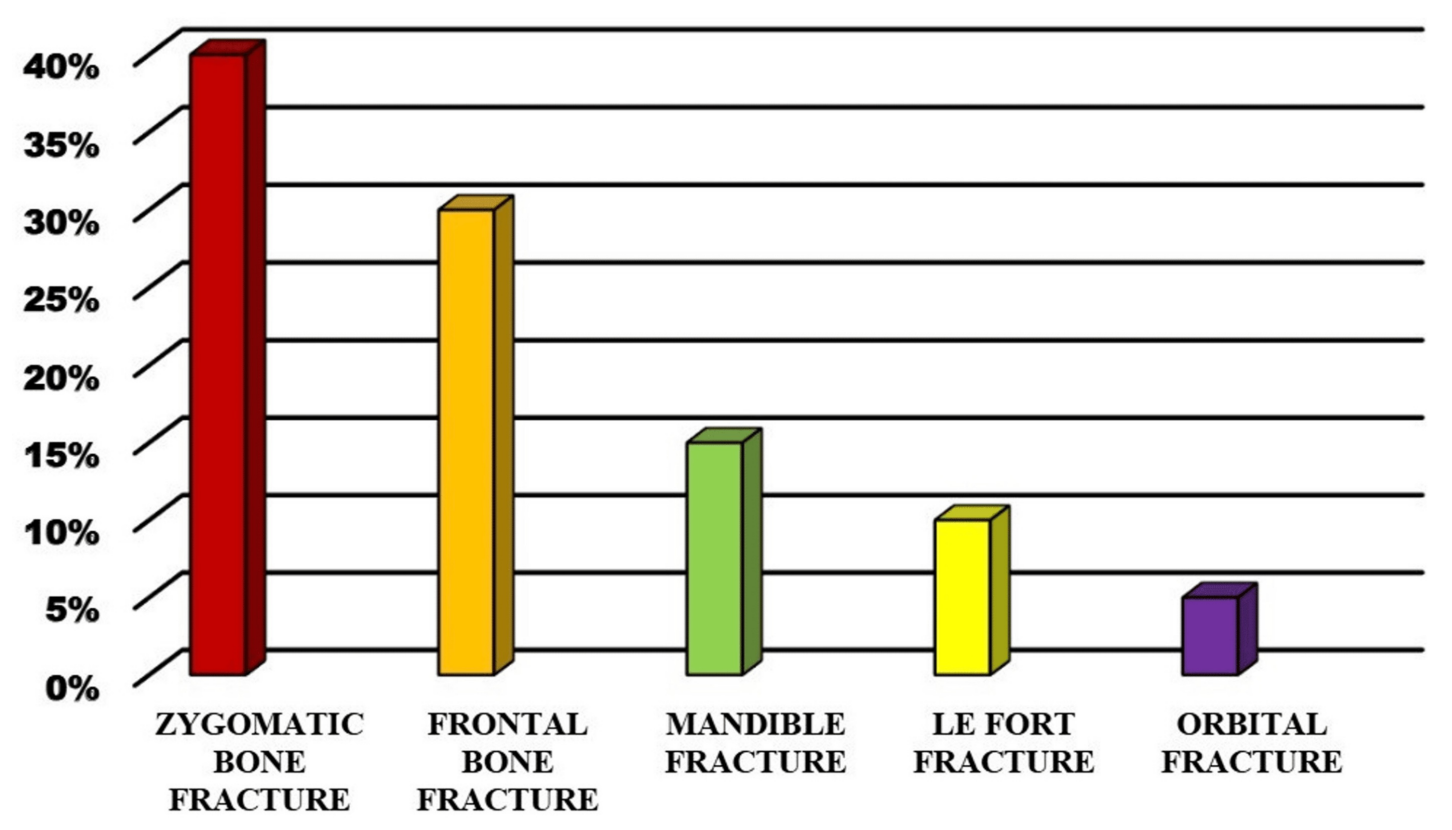
Different types of maxillofacial injuries (in %)

Facial fractures involving the zygomatic bone were the most commonly associated with brain injury, found in 40% of patients with brain trauma. Frontal bone fractures were the second most common, present in 30% of brain injury cases. Mandibular fractures were associated with brain injury in 15% of cases, while Le Fort fractures and orbital fractures accounted for 10% and 5%, respectively (Figure 4). Patients with zygomatic and frontal bone fractures were more likely to have severe brain injuries, including intracranial hemorrhage and skull fractures, compared to those with mandibular or nasal fractures (p < 0.01).

Glasgow Coma Scale and outcome

Patients with brain injuries had significantly lower GCS scores on admission (mean score: 8) compared to those without brain injury (mean score: 14). A strong correlation was found between lower GCS scores and prolonged hospital stays, as well as the need for intensive care and mechanical ventilation.

Treatment and surgical management

Surgical intervention was performed for maxillofacial fractures in 176 patients, which represents 80% of the total. Nevertheless, in 20% of patients with brain injuries, the surgical treatment of facial fractures was postponed because immediate neurosurgical procedures were necessary, such as craniotomies and management of intracranial pressure. In these situations, collaboration between neurosurgeons and maxillofacial surgeons was essential.

Complications and mortality

Individuals with brain injuries experienced a notably increased incidence of complications, such as post-traumatic seizures, infections, and the requirement for additional surgeries. Within the study group, the overall death rate was 5%, with brain injury identified as the leading cause of death in every instance that resulted in fatalities.

## Discussion

This research emphasizes the notable link between maxillofacial fractures and traumatic brain injuries, especially in instances of zygomatic and frontal bone fractures. These results align with previous studies, which suggest that the closeness of these facial bones to the skull heightens the likelihood of brain damage. Prompt recognition of brain injuries in patients with maxillofacial trauma is essential, as postponements in treatment may result in unfavorable results.

One significant obstacle in treating patients with concurrent maxillofacial and brain injuries is determining the order of care. When a brain injury is critical, surgical treatment for the brain is prioritized, which can postpone maxillofacial procedures and heighten the risk of complications such as malunion or infection. Multidisciplinary teams need to collaborate effectively to reconcile the immediate need for brain protection with the necessity of addressing facial fractures.

Dentoalveolar fractures, nasal bone fractures, mandibular fractures, maxillary fractures, frontal bone fractures, naso-orbito-ethmoid fractures, panfacial fractures, and penetrating injuries are among the range of maxillofacial injuries that present in the trauma unit. Concomitant cerebral, pulmonary, intra-abdominal, or extremities injuries are possible in patients with maxillofacial fractures [15].

Although nature has safeguarded the brain with a robust helmet of thick bone, the facial bones that are important for vision, taste, smell, chewing, and aesthetics are relatively delicate [16]. It has been suggested that the face may serve a protective role for the brain similar to how an airbag shields the chest in a car accident. In various countries, cranial injuries have been identified as the most frequently occurring concomitant injuries in patients with maxillofacial trauma, which encompasses head injuries, intracranial bleeding, closed head traumas (such as brain contusions or lacerations), and skull fractures [17,18]. Numerous studies have indicated a strong correlation between maxillofacial fractures and intracranial injuries [19].

In general, symptoms such as forgetfulness, vomiting, emesis, unconsciousness, or a low Glasgow Coma Scale (GCS) score are significant indicators of a possible cranial injury. Nevertheless, head injuries in patients with maxillofacial trauma may be observed without these symptoms [20,21]. A potentially fatal factor that raises mortality in patients with maxillofacial trauma is the presence of brain injuries [20]. However, research has not yet conclusively established the precise connections between various kinds of facial fractures and brain damage, according to Fonseca et al. [22].

The overconsumption of alcohol is closely linked to facial trauma [23]. In our research, 80% of the individuals examined were under the influence of alcohol. The increased intake of alcohol and substance misuse has further contributed to the statistical rise of road traffic accidents (RTAs) in our nation. Alcohol affects decision-making, can incite aggression, frequently leads to violence between individuals, and is a significant contributor to car accidents.

This research indicates that the primary factor contributing to head injuries is road traffic accidents (RTAs). This suggests that there is a necessity for the enforcement of strict road safety regulations, which should encompass speed restrictions, laws requiring the use of helmets and seat belts, and the development of well-designed and high-quality road infrastructure.

Furthermore, this research indicates that it is a mistake to consider fractures of the facial skeleton as isolated incidents, because, as evidenced in this study, they are linked with more severe, and at times life-threatening, head injuries that necessitate a comprehensive assessment upon presentation. The surgical management of patients who have sustained both head and neck trauma is highly personalized and is influenced by several factors, including the cause of the injury, accompanying injuries, the patient's age, and the potential for an interdisciplinary approach. This study leads to the conclusion that understanding the associated injuries promotes appropriate care and expedited recovery. It is only through a multidisciplinary and coordinated strategy that optimal success can be achieved in treating patients with facial fractures and related injuries.

## Conclusions

Maxillofacial injuries frequently occur in road traffic accidents (RTAs) and various other types of trauma, as the head and face often become the initial parts of the body to act as secondary missiles during ejection from a vehicle. During motor vehicle collisions, the forces acting on the head can exceed several times that of gravity. Head injuries tend to have more severe consequences, typically linked with these events, necessitating prompt medical attention. This prospective study found a notable correlation between head injuries and maxillofacial trauma. The likelihood of sustaining a head injury increased with the number of maxillofacial fractures and decreased Glasgow Coma Scale (GCS) scores. Therefore, every patient with a maxillofacial fracture should undergo thorough clinical and radiological evaluation to exclude any potential head injuries and reduce the risk of mortality. Timely diagnosis and prompt treatment are crucial for minimizing both morbidity and mortality, particularly in preventing traumatic brain injury (TBI), as even brief episodes of hypoxia and edema can result in considerable lasting neurological impairments.

This study highlights the critical need for prompt and precise identification of brain injuries in individuals with facial trauma. Fractures of the zygomatic and frontal bones show a notable correlation with brain injuries; thus, patients with these fractures ought to receive comprehensive neurological assessment and imaging. The findings support a multidisciplinary approach involving both neurosurgical and maxillofacial teams to improve patient outcomes. Such coordinated care is essential in managing complex trauma cases where the risk of neurological damage is significant.
